# Mental health and suicidality in glioblastoma patients in germany – a multicenter cross-sectional observational study protocol (SHINE-GLIO Study)

**DOI:** 10.1186/s42466-026-00520-5

**Published:** 2026-08-06

**Authors:** Mareike Rutenkröger, Maximilian Scheer, Niklas Benedikt Pepper, Felix Ehret, Franziska M. Ippen, Teresa Schmidt, Jana Grieger, Giorgio Cappello, Leon Jekel, Lazaros Lazaridis, Ulrich Sure, Laurèl Rauschenbach, Yahya Ahmadipour, Christoph Kleinschnitz, Lasse Dührsen, Joachim Peter Steinbach, Heidrun Golla, Corinna Seliger-Behme, Christoph Oster, Sied Kebir

**Affiliations:** 1 Department of Medical Psychology, University Medical Center Hamburg-Eppendorf, Hamburg, Germany; 2 Department of Neurosurgery, University Hospital Heidelberg, Heidelberg, Germany; 3 Department of Radiation Oncology, University Hospital Muenster, Muenster, Germany; 4 Department of Radiation Oncology, Charité – Universitätsmedizin Berlin, Corporate Member of Freie Universität Berlin and Humboldt-Universität Zu Berlin, Berlin, Germany; 5 German Cancer Consortium (DKTK), Partner Site Berlin, a partnership between DKFZ and Charité – Universitätsmedizin Berlin, Berlin, Germany; 6 Department of Neurology, University Hospital Heidelberg, Heidelberg, Germany; 7 German Cancer Research Center (DKFZ), Clinical Cooperation Unit Neuropathology, German Consortium for Translational Cancer Research (DKTK), Heidelberg, Germany; 8 National Center for Tumor Diseases (NCT), NCT Heidelberg, a partnership between DKFZ and University Hospital Heidelberg, Heidelberg, Germany; 9 Department of Neurology and Center for Translational Neuro- and Behavioral Sciences (C-TNBS), Division of Clinical Neuro-Oncology, University Medicine Essen, University of Duisburg-Essen, Essen, Germany; 10 Department of Neurology, University Hospital Knappschaftskrankenhaus Bochum, Ruhr University Bochum, Bochum, Germany; 11 Department of Neurosurgery and Spine Surgery and Center for Translational Neuro- and Behavioral Sciences (C-TNBS), University Medicine Essen, University of Duisburg-Essen, Essen, Germany; 12 German Cancer Consortium (DKTK), Partner Site Essen/Düsseldorf, a Partnership Between Deutsches Krebsforschungszentrum (DKFZ) and University Hospital Essen, DKFZ-Division Translational Neuro-Oncology at the West German Cancer Center (WTZ), Essen, Germany; 13 Department of Neurosurgery, University Medical Center Hamburg-Eppendorf, Hamburg, Germany; 14 Dr. Senckenberg Institute of Neurooncology, University Hospital, Goethe University, Frankfurt, Frankfurt, Germany; 15 Department of Palliative Medicine, University Medical Center Göttingen, Göttingen, Germany

**Keywords:** Glioblastoma, Suicidality, Depression, Anxiety, Patient-reported outcomes, Germany

## Abstract

**Introduction:**

Glioblastoma is an aggressive primary brain tumor with poor survival. Depressive and anxiety symptoms and suicidality are common and impair quality of life and care. Prospective multicenter data using validated measures of suicidal ideation and behavior in German glioblastoma cohorts are scarce. We aim to quantify psychiatric symptom burden and suicidality and examine associations with demographic, clinical, and tumor-related characteristics.

**Methods:**

SHINE-GLIO is a multicenter, cross-sectional observational study at German neuro-oncology centers. Adults with newly diagnosed isocitrate dehydrogenase wild-type glioblastoma after primary radiotherapy and before first recurrence are eligible. Exclusion criteria include clinically significant aphasia, other malignancies, and known tumor predisposition syndromes. At one outpatient visit, participants undergo physical examination and complete validated self-report questionnaires assessing suicidality (SSEV), depressive symptoms (PHQ-8), anxiety (GAD-7), social support (SUCE−4), and quality of life (EORTC QLQ-C30 and QLQ-BN20). Tumor characteristics, clinical history, and concomitant medications are extracted from medical records. Prespecified regression models will examine associations with suicidality while accounting for demographic, clinical, and tumor-related factors.

**Perspective:**

This study will provide a standardized multicenter assessment of suicidality and related psychiatric symptoms in German patients with glioblastoma. The findings may inform screening and referral pathways for mental health care.

**Trial registration:**

ClinicalTrials.gov identifier: NCT07409584. First posted prospectively on February 12, 2026.

## Introduction

Glioblastoma is an aggressive primary brain tumor with an unfavorable prognosis. In unselected contemporary cohorts, median overall survival is generally between 12 and 18 months, whereas selected molecular subgroups may experience substantially longer survival [1–3]. It remains among the solid tumors with the shortest survival despite multimodal therapy. Management of newly diagnosed glioblastoma typically includes maximal safe tumor resection, followed by combined radiotherapy and temozolomide chemotherapy. In selected patients, treatment may be supplemented with tumor-treating fields. Since the introduction of radiotherapy plus temozolomide in 2005, only a small number of randomized phase III trials have demonstrated an overall survival benefit in newly diagnosed glioblastoma, largely driven by alkylating regimens rather than targeted or immune therapies [1]. Nearly all patients ultimately develop disease progression despite initial therapy.

At recurrence, management is individualized and may include repeat surgery, reirradiation, and systemic agents, but evidence is heterogeneous and no universally accepted overall-survival–prolonging standard exists [4]. Given the poor prognosis and high neurological morbidity, a glioblastoma diagnosis can profoundly disrupt emotional well-being, physical function, and social roles [5, 6]. Compared with many other cancers, brain tumors uniquely threaten cognition, communication, behavior, and autonomy, complicating decision-making and social participation [7, 8]. Psycho-oncological consultations frequently reveal high psychological burden, with many patients expressing concerns about death and a strong need for medical information [9]. Hence, patients with glioma frequently experience psychiatric comorbidities such as depression and anxiety, which are associated with diminished quality of life and may negatively affect treatment adherence and survival. By comparison, suicidality remains understudied and is rarely assessed in a standardized manner [10–13]. Depressive symptoms may reflect direct neurobiological and treatment-related effects (e.g., lesion location, inflammation, corticosteroid exposure) and/or a psychological response to diagnosis and functional loss [11]. Longitudinal trajectories of depression and anxiety after surgery are heterogeneous, with some patients improving transiently and others worsening as treatment burden and neurological disability accumulate [14, 15].

Suicide mortality is elevated in cancer patients compared with the general population, with the magnitude of risk varying by cancer type and being particularly high in poor-prognosis tumors [16]. Data on suicidality in glioblastoma specifically are limited. Large population-based analyses indicate higher suicide mortality after cancer diagnosis, with risk varying by tumor type and time since diagnosis; poor-prognosis central nervous system (CNS) malignancies consistently rank among higher-risk groups [17]. Existing literature suggests that suicidality in brain tumor populations likely reflects interacting neuroanatomical, treatment-related, inflammatory, and psychosocial factors, although evidence is heterogeneous and often observational. While inflammatory biomarkers such as tumor necrosis factor alpha (TNF-α) and interleukin-6 (IL-6) have shown associations with suicidal ideation in psychiatric samples, evidence regarding their significance in glioblastoma is limited, and tumor-specific studies are needed to clarify their role [18–21].

Registry-based analyses report low absolute numbers of death by suicide in malignant brain tumor patients, but interpretation is limited by ascertainment, follow-up time, and the fact that suicidal ideation is rarely captured in such datasets. In registry studies, higher suicide mortality has been associated with demographic factors (e.g., male sex and older age) and tumor-related characteristics (e.g., supratentorial location), although findings may reflect confounding and may not generalize across healthcare systems and populations. Adjusted registry models suggest that suicide mortality may be highest within the first year after diagnosis and is associated with age, sex, and tumor location, although causality cannot be inferred [22]. Another U.S. population-based analysis likewise reported that suicide deaths are rare events in brain tumor registries and appear more frequent in older male patients and in glioblastoma, underscoring the need for studies that directly measure suicidal ideation and behavior [23]. A systematic review reported that suicidal ideation is substantially more common than suicide attempts in brain tumor populations [24], but estimates vary widely across studies due to heterogeneous measurement tools, timepoints, and tumor subtypes. Across heterogeneous studies, suicidal ideation and behavior have been associated with depressive and anxiety symptoms, functional impairment, and selected demographic and tumor-related characteristics, although endpoint definitions differ substantially.

However, suicide mortality estimates from administrative registries are vulnerable to misclassification and under-ascertainment, and their generalizability to contemporary Central European healthcare settings remains uncertain. Most datasets also do not assess how many individuals experienced significant suicidal ideation without progressing to recognized suicide.

Diagnosis of high-grade glioma often causes significant psychosocial stress in affected patients. Even structured distress screening strategies have shown limited impact on downstream psychosocial care uptake, highlighting implementation gaps relevant to mental health risk detection. The GLIOPT study investigated whether screening for distress based on physician–patient conversations was improved in the intervention group compared with the control group, which completed only the Distress Thermometer. In that trial, the endpoint focused on the number of patients with impaired emotional functioning who received specialized psychosocial care, rather than on suicidality-specific outcomes [25]. This underscores that generic distress screening alone may be insufficient to trigger appropriate psychosocial interventions, supporting the need for suicidality-specific assessment in glioblastoma.

Functional impairment in intracranial tumor patients may coincide with greater psychological burden, supporting the inclusion of performance status as a potential correlate of suicidality. In a German longitudinal cohort, 21.5% of participants reported suicidal thoughts at least once during follow-up, although estimates are limited by tumor heterogeneity and measurement approach. Reported suicidal ideation fluctuated over follow-up timepoints, with peaks in some subgroups, but subgroup-specific estimates are difficult to generalize to glioblastoma. That study suggested that suicidal thoughts can occur across different intracranial tumor histologies, alongside reduced health-related quality of life, although generalizability is limited [26]. Against the backdrop of these findings, our planned study will examine suicidality in a multicenter design, focusing on glioblastoma patients, with slightly modified endpoints in light of the current WHO CNS classification.

In summary, depression, anxiety, and suicidality constitute clinically relevant and potentially under-recognized comorbidities in glioblastoma care. Population-based studies suggest increased suicide mortality in malignant brain tumor populations, while suicidal ideation is likely far more prevalent yet less systematically measured. Nevertheless, available data remain limited, and prospective studies are scarce.

## Methods

### Aim of the study

This multicenter, cross-sectional observational study aims to assess associations between suicidality and clinical and psychological variables in patients with glioblastoma. Validated questionnaires will assess anxiety, depression, disease-specific health-related quality of life, and social support. The primary endpoint is suicidality during the past four weeks, measured at the single study visit using the SSEV score. Secondary endpoints include anxiety symptoms (GAD-7), depressive symptoms (PHQ-8), health-related quality of life (EORTC QLQ-C30 and EORTC QLQ-BN20), and social support (SUCE−4). The findings are intended to inform the design of future supportive-care interventions for depression, anxiety, and suicidality in glioblastoma.

### Study design

Approval for this prospective, multicenter observational study was granted by the Ethics Committee in Essen, Germany (approval no. 25—12,746-BO), and all participating sites obtained local ethical approval before enrolling participants. The study will recruit 180 patients diagnosed with glioblastoma. Manuscript preparation followed the STROBE (Strengthening the Reporting of Observational Studies in Epidemiology) reporting guidelines. To ensure feasibility and reduce burden on participants, all assessments are scheduled within one outpatient visit. The study is conducted in accordance with the Declaration of Helsinki and applicable national legislation.

### Study procedures

Patients can be included in the study after completion of primary radiotherapy and before the first recurrence (local MRI assessment according to RANO 2.0 [27]). After being informed about the study objectives during a physician consultation, patients provide written informed consent prior to enrolment. Questionnaires are completed independently by participants without the presence of relatives. During a single outpatient visit, trained staff perform a standardized clinical and neurological assessment, including Karnofsky Performance Status. Participants also complete the predefined self-report instruments listed in Table 1, which are administered under strictly standardized self-completion conditions. If, when completing the SSEV questionnaire, at least one question is answered with “less than once a week” or more often, further evaluation will be conducted during a medical consultation on the same day, and based on its results the patient will be referred to psycho-oncological care. Patient data are obtained from medical records by a clinical investigator based on a standardized case report form (demographics, diagnosis/tumor data, previous cancer treatment, MRI findings, medical history, medication, vegetative symptoms, substance use, family history of cancer) and the study visit. The recruitment period runs from March 18, 2026, through March 17, 2028. As the primary study center, Essen is expected to recruit approximately 60 participants, or 30 per year. The remaining approximately 120 participants in the planned total sample of approximately 180 participants, including anticipated dropouts, will be recruited across the other participating centers. Each center is expected to recruit approximately 10–20 participants over the two-year recruitment period. The regular follow-up of patients with glioblastoma at these specialized centers provides an established population from which potentially eligible participants can be screened. Based on these ongoing clinical follow-up structures and the multicenter design, recruitment of the planned study population by March 17, 2028, is considered feasible.

**Table 1:** Overview of study endpoints

Endpoints	Measure	Brief description
*Primary*		
Suicidality	Scale for Suicidal Experience and Behavior (SSEV) [28]	The SSEV is a 9-item self-report questionnaire assessing suicidal thoughts, impulses, intentions, plans, and attempts within the past four weeks. The primary outcome will be a continuous composite score based on the first six items (range 0–18), with higher scores reflecting greater suicidal ideation severity. The 6-item score was selected to ensure a standardized and sensitive measure across participants Secondary and sensitivity analyses will include a dichotomized version of suicidal ideation (score ≥1 on the 6-item scale, indicating presence vs. absence). Lifetime suicide attempts will be assessed separately using a single yes/no item and treated as a secondary outcome
*Secondary*		
Depressive symptoms	Patient Health Questionnaire−8 (PHQ-8) [30]	Depressive symptoms are measured by the validated German PHQ-8, with responses on a 0–3 Likert scale (“0-not at all” to “3-nearly every day”). Scores will be analyzed continuously and categorized into standard severity levels (minimal to severe). The PHQ-8 was preferred over the PHQ-9 to avoid inclusion of a suicidality item and thus prevent overlap with outcome measures in suicidality analyses
Anxiety symptoms	Generalized Anxiety Disorder-7 (GAD-7) [31]	Anxiety is assessed using the GAD-7, with standard response options from 0 (“not at all”) to 3 (“nearly every day”) and established cutoffs for mild (≥ 5), moderate (≥ 10), and severe (≥ 15) anxiety
Health-related quality of life (disease-specific)	EORTC QLQ-C30 [32] and EORTC QLQ-BN20 [33]	Quality of life is assessed using the EORTC QLQ-C30, a 30-item instrument evaluating general cancer-related quality of life. In addition, the EORTC QLQ-BN20 is used to capture brain tumor–specific symptoms and functional impairments, including visual problems, communication difficulties, future uncertainty and motor dysfunction
Social support	Short Questionnaire on Social Support in Chronic Illness (SUCE−4) [29]	The SUCE−4 is a brief instrument assessing perceived social support in individuals with chronic illness. All four items are assessed on a 5-point Likert scale and coded numerically (0 to 4 from ‘no’ to ‘yes’), with any assistance during completion and any missing items documented according to prespecified rules. The “social support” score ranges from 0 to 16 and is the sum of all four items
Physical impairments/disabilities	Physical Examination / Medical Records	Physical impairments and disabilities are assessed through a standardized clinical and neurological examination and review of the medical records. Relevant impairments, including motor, sensory, visual, language, and other neurological deficits, as well as their impact on functional status, will be documented
Tumor location	Medical records/MRI	The MRI closest to the study visit (within a prespecified time window) will be used to code tumor location using predefined anatomical categories (cerebellum, brainstem, temporal, frontal, parietal, occipital, basal ganglia, or spinal)
Marital status	Social History	Marital status is recorded during the medical interview as part of the social history. The interview follows standard demographic conventions [34]

### Outcome measures

#### Medical history/assessment of concomitant medication and comorbidities

A medical history covering recorded previous illnesses (both psychiatric and somatic), past surgeries, allergies, intolerances, current and past medications, use of stimulants or addictive substances based on the participant’s statement, family and social history, as well as subjective side effects of previous tumor-specific treatments based on the participant’s statement and medical records will be obtained during the study visit.

#### Physical examination

A standardized neurological examination will be performed at the study visit, covering motor, sensory, cerebellar, cranial nerve, and language domains, with key deficits documented in predefined categories for subsequent analyses.

#### Karnofsky performance score

Trained clinicians will rate the Karnofsky Performance Status at the study visit using standardized anchor descriptions, and the timing of rating (before questionnaire review) will be prespecified to minimize observer bias.

#### MRI

MRIs are acquired as part of routine care and will be evaluated once for the purposes of this study. The study-specific analysis is conducted without knowledge of the questionnaire results. The MRI closest to the study visit (within a prespecified time window) will be used to code tumor location using predefined anatomical categories, and tumor extent will be operationalized using imaging-defined measures (e.g., contrast-enhancing core location and FLAIR hyperintensity involvement), with explicit rules for multilobar or multifocal disease.

*Patient-Reported Outcome Measures.* Table 1 gives an overview of the study endpoints and all measurement instruments used.

### Eligibility criteria

The following criteria apply for inclusion in the study: newly diagnosed glioblastoma, isocitrate dehydrogenase (IDH) wild-type (CNS WHO Classification 2021) after completion of primary radiotherapy and before first recurrence (local MRI assessment according to RANO 2.0 [27]), and an age of at least 18 years. Exclusion criteria include language barriers or clinically significant aphasia as assessed by the investigator, a known tumor predisposition syndrome such as Li-Fraumeni syndrome or Cowden syndrome, a history of other malignant tumors, and recurrent disease. Eligibility is assessed during a preliminary screening conducted by a board-certified physician with expertise in neuro-oncology as part of the routine outpatient consultation. Participants are recruited through neuro-oncology centers and departments in Germany. No financial compensation is provided for participating centers or patients. Likewise, insurance coverage for participants is not offered.

### Sample size calculation

A sample size calculation has been performed for a multiple linear regression analysis with ten candidate predictor domains: marital status (single, married, divorced, or widowed), age (in years), physical impairment (Karnofsky Performance Status), sex (male/female), depression (PHQ-8), anxiety (GAD-7), social support (SUCE−4), health-related quality of life (EORTC QLQ-C30 and EORTC QLQ-BN20), time since diagnosis (in days) and tumor location (brainstem, frontal, parietal, cerebellum, temporal, occipital, basal ganglia, or spinal). An exploratory sample size calculation was conducted for the planned multiple linear regression analysis, including approximately 20 regression parameters, accounting for the use of dummy coding for categorical variables with more than two levels. Based on a medium effect size assumption (f^2^ = 0.15; corresponding to R^2^ ≈ 0.13), an alpha level of 0.05, and a desired statistical power of 0.80, the required sample size was estimated at 157 participants using G*Power. This calculation should be interpreted as exploratory, particularly given the uncertainty regarding the magnitude of the expected effects and the possibility that effects may be smaller than anticipated. Therefore, all analyses will report confidence intervals and effect size estimates alongside significance tests to allow for an informed interpretation of the findings, irrespective of whether medium-sized effects are observed. To account for an anticipated attrition and data loss rate of approximately 15% (e.g., due to incomplete questionnaires, missing covariate data, or exclusions during data cleaning), the planned recruitment target was increased from 157 to 180 participants.

#### Statistical analyses

Associations between suicidality and candidate predictors will be examined using regression models appropriate to the distribution of the SSEV outcome, defined as the continuous 6-item SSEV composite score assessing suicidal ideation during the past four weeks. Depending on distributional properties, linear regression models will be used for approximately continuous and symmetric outcomes, while generalized models will be considered in case of marked skewness or zero inflation. All assumptions (including linearity, homoscedasticity, and distribution of residuals) will be assessed and reported where applicable. Prior to multivariable modeling, descriptive statistics and bivariate correlations (Pearson or Spearman, depending on scale properties and distribution) will be computed to describe the data structure. These analyses are purely exploratory and will not be used for variable selection. Multicollinearity will be assessed using variance inflation factors (VIF).

As secondary and sensitivity analyses, additional models will be fitted using (a) a dichotomized suicidal ideation outcome (presence vs. absence of suicidal ideation, defined as a 6-item SSEV score ≥ 1) and (b) lifetime suicide attempts assessed by the dedicated dichotomous SSEV item. These analyses will include the same set of predictors and are intended to assess the robustness of findings across alternative operationalizations of suicidality. All analyses are considered exploratory and hypothesis-generating and are not intended to establish causal relationships.

Given the exploratory nature of the study, all prespecified candidate predictors will be entered simultaneously into a multivariable model without prior univariable screening. To reduce overfitting and improve the stability of estimates in the presence of a relatively high number of predictors, penalized regression (ridge regression with tuning via cross-validation) will be applied. Model performance will be internally validated using bootstrap resampling (e.g., 5,000 resamples) to estimate and correct for optimism in performance metrics. Regression coefficients with 95% confidence intervals will be reported, along with model performance measures such as R^2^ and root mean squared error (RMSE) for the continuous outcome.

Missing data will be reported for all variables. Missingness patterns will be explored descriptively, and multiple imputation will be used to handle missing values under the “missing at random” (MAR) assumption. All predictors included in the analysis model will also be included in the imputation model.

## Perspective

This multicenter observational study with a single cross-sectional assessment will estimate the past-4-week frequency and severity of suicidality [28] and examine cross-sectional associations with demographic, clinical, and tumor characteristics in adults with isocitrate dehydrogenase (IDH) wild-type glioblastoma enrolled between initial diagnosis and first recurrence. Depressive and anxiety symptoms are common in glioblastoma. They are associated with shorter overall survival and poorer health-related quality of life in meta-analytic evidence, whereas suicidality in brain tumor populations appears elevated yet remains incompletely characterized beyond registry-based endpoints. However, the literature is dominated by retrospective registry analyses and small single-center cohorts with variable definitions and observation windows for suicidality, often without standardized assessment of suicidal ideation and attempts. In particular, prior datasets often lack granular neuro-oncologic and treatment variables (e.g., functional status, corticosteroid exposure, antiseizure medication, extent of resection, and molecular markers) alongside standardized patient-reported outcomes, limiting confounder adjustment and interpretability.

The primary objective is to model cross-sectional associations between suicidality (SSEV) and depressive symptoms (PHQ-8), anxiety symptoms (GAD-7), and prespecified demographic, clinical, and tumor characteristics. As secondary objectives, the study aims to characterize the distribution of depressive and anxiety symptom severity and to explore how suicidality and overall symptom burden relate to tumor location, functional status as measured by the Karnofsky Performance Score, and perceived social support (SUCE−4) [29].

To achieve these goals, the protocol combines validated patient-reported outcome measures with standardized neurological examination and structured abstraction of treatment and imaging variables across centers.

The study is designed to minimize patient burden by integrating all procedures into a single outpatient visit, while ensuring that data are collected in a standardized manner across multiple centers using a standardized case report form and training before delegation.

From an operational standpoint, the study addresses the practical challenges of recruitment, cognitive impairments, language limitations, and data completeness. Trained staff oversee standardized questionnaire completion without influencing responses to ensure high-quality, reliable data. The study design also allows for the exploration of psychosocial variables underrepresented in routine neuro-oncological practice, such as perceived social support.

The resulting multicenter dataset will support empirically grounded hypotheses and candidate correlates of suicidality that may inform pragmatic screening pathways and require validation in independent prospective cohorts. If replicated, the identified associations can inform the design of targeted, testable psycho-oncological interventions and referral algorithms, which will require prospective interventional studies to demonstrate improvements in patient-centered psychosocial outcomes.

## Conclusions

This multicenter cross-sectional observational study aims to explore potential clinical, demographic, and psychological correlates of suicidality in patients with glioblastoma treated in German neuro-oncology centers. By identifying factors associated with suicidality and related outcomes, the study seeks to generate hypotheses for future research and to improve the understanding of suicidality in this patient population.

Given the exploratory design, the findings are not intended to establish causal relationships but may help inform the development of future prospective studies and support the identification of patients at increased psychological risk. Suicidality and related psychological symptoms remain highly relevant for patient quality of life in neuro-oncology and warrant further systematic investigation.

## Data Availability

No datasets were generated or analysed during the current study.

## Declarations

### Permission for reproduction of material from other sources

No previously published material requiring permission is reproduced.
